# The General Medical Council

**Published:** 1896-12-12

**Authors:** 


### THE GENERAL MEDICAL COUNCIL.

The sixty-first session of the General Medical Council was commenced on November 24th, and brought to a termination on December 1st. The most important business of the session arose out of the renewed application of the Apothecaries' Hall, of Dublin, to be provided with assistant examiners by the Council, and thus to be rendered an independent qualifying body for the whole of the United Kingdom. In order to render the question quite intelligible it is necessary to enter into some preliminary explanations.

The Medical Act of 1886 provided that no one should be permitted to register, as a medical practitioner, until he had passed an examination in medicine, surgery, and midwifery; so that, from thenceforward, the license given by a body legally qualified to examine in only one or only two of these subjects was rendered of no avail. In England, for example, the Royal College of Surgeons was only authorised to grant licenses in surgery; and the Society of Apothecaries was only authorised to grant licenses in medicine and midwifery. In order to prevent the practical extinction of bodies so situate, the Act provided that for the purposes of conducting examinations they might combine with any other bodies capable of supplying their deficiencies; or that, if unable so to combine, they might apply to the Medical Council to appoint assistant examiners in the subjects as to which they were deficient. The Medical Council had discretion either to grant or to refuse such an application; but, in case of refusal, an appeal was given to the Privy Council, with which the final decision would rest. In England the Royal Colleges of Physicians and of Surgeons combined to form a Conjoint Board, from which the Society of Apothecaries was excluded; and the Society applied to the Medical Council for assistant examiners in surgery, so as to render its board complete. These were given by the Council, with the result that the Society was permanently re-established as a body empowered to confer a complete medical and surgical qualification, and to place its licentiates upon the medical register by virtue of this qualification alone. In Dublin, as in London, there was a College of Physicians, a College of Surgeons, and an Apothecaries' Hall, but the order of events there was different. The College of Surgeons formed two combinations, one with the College of Physicians, and another with Apothecaries' Hall, so as to form two Conjoint Boards, each fully qualified to admit its licentiates to the register. The inspections and visitations of the Medical Council disclosed the fact that the examinations of the Conjoint Board formed by the Irish College of Surgeons and the Dublin Hall were anything but satisfactory, especially upon their medical side; and, finally, the Council reported to the Privy Council that these examinations were "insufficient." In the meanwhile, the College of Surgeons, dissatisfied with its own position in relation to them, had given notice to withdraw from its combination with the Hall, and hence the Privy Council, as the impugned board was about to die a natural death, did not think it necessary to take any action in consequence of the Medical Council's report. The Dublin Hall, however, was unwilling to cease to exist as a qualifying body, and made application to the Medical Council for assistant examiners in surgery. This application the Council refused to grant, being apparently influenced by the consideration that the medical side of the examination would still be under the control of the Hall, and that it was this side which had been adversely reported upon by the inspectors. The Hall appealed to the Privy Council, by which the question was adjourned, in the hope that a *modus vivendi* might be discovered, and the Irish College of Surgeons was approached with reference to the possibility of a fresh combination. The College, however, declined even to consider the matter; and so, at the session of the Council which has just terminated, the Hall made application for assistant examiners in medicine as well as in surgery. The Council refused the application by an overwhelming majority (23 votes to 2, with one absentee and four members not voting), so that the Hall will cease to exist as a qualifying body unless this decision is reversed on appeal by the Privy Council. That it will be reversed, even if the appeal of the Hall be prosecuted, seems very improbable. The Privy Council would hardly desire to assume the responsibility of supporting a medical examining board which had been so emphatically condemned by medical authority; and the Hall does not seem to exercise any such influence in political circles as might lead to its rehabilitation on account of considerations among which medical requirements would have no place. Whatever may be the ultimate issue, the occasion is noteworthy as being the first on which any medical examining body has stood in real peril of extinction.

The Council received some reports on the powers of various qualifying bodies to withdraw licenses or degrees from persons who had been guilty of crime or misconduct; and the whole subject was referred to the Executive Committee for consideration and further report.

The Education Committee reported in favour of raising the standard of preliminary examinations in general knowledge which are demanded from persons desiring to be admitted as medical students; and it was resolved that a scheme for this purpose should be brought up next year, with a view to its being adopted and brought into force on January 1st, 1900, so as to give students and teachers the necessary time for preparation.

Three medical practitioners were charged with improperly employing unqualified assistants in such a way that these persons were under no supervision, and were enabled to assume professional responsibilities as if they were qualified. All the charges were found to be proven, and all were adjourned to the next session, so as to give the defendants time to abandon the practices complained of. An adjourned charge of the same kind against a dentist was disposed of. The defendant showed that he had obtained a qualified assistant, and promised not to repeat the offence, on which the Council decided to take no further action in the matter. On the report of the Executive Committee, the names of Mr. George Davidson and of Mr. Neville Holland, which had been erased from the Register in 1894 for the offence of "covering" unqualified persons, were ordered to be restored; and other applications for restoration were refused or deferred.

A report was presented by the Finance Committee showing that the Council had now acquired the freehold of the premises in which its offices are situate, as well as that of the adjoining house in Hanover Square, and that this had been done on advantageous terms.

A largely-signed memorial in favour of the addition of shorthand writing, as an optional subject, to the preliminary examination, having been presented to the Council, and reported upon by the Education Committee, it was resolved that examiners should be empowered to assign marks for this subject, not exceeding 5 per cent. of the total obtainable under the head of "English language, including grammar and composition." It was felt that this would encourage proficiency in shorthand as a method of writing, while it would avoid placing it, as an "optional" subject, on the level of Greek, or of a modern language.

During the hearing of the charges of "covering," attention was drawn to the extent to which, in some parts of England, the registrars of deaths were in the habit of accepting certificates of the causes of death from unqualified persons; and it was resolved to ask the President of the Local Government Board to receive a deputation on the subject.

A very large question relating to the conduct of the final examination for a licence to practice was raised last session by Mr. Teale, and the Examinations Committee reported upon his proposals. After a prolonged discussion, the report was referred back to the committee for further consideration, and to be presented again at the May session of the Council. Another important debate was founded upon the reports of a committee on qualifying examinations in dentistry; but this also was adjourned until May.

Of the thirty members of the Council, five, as is well known, are elected by the votes of medical practitioners: three in England, one in Scotland, and one in Ireland; and the term of office of these "direct" representatives will expire at the end of the present year. In Ireland, we believe, there is no opposition to the present representative; and in Scotland none that need be seriously considered; but two of the English representatives, Sir Walter Foster, M.P., and Mr. Wheelhouse, will not seek re-election, and the third, Dr. Glover, will not be suffered to walk over. There are ten candidates in the field for the three seats, and one result of this has been that, during the whole of the session just concluded, there has been a good deal of electioneering in the air. The controversies hence arising, however, have been of such merely temporary interest that it is not worth while to occupy space in recording them, more especially as, at the time of writing, all the voting papers have been delivered, and the contest is practically decided. A special meeting of the English Branch Council will be held on Friday, the 11th instant, for the purpose of officially declaring the result. Whoever may be elected, it will be long before the new members can hope to attain to the positions held in the Council by those who have withdrawn from it; and the concluding business of the past session was to carry by acclamation the following resolution, which was proposed by Mr. Brudenell Carter, seconded by Mr. Teale, and supported by Sir William Turner: "That in view of the approaching retirement of Sir Walter Foster and Mr. Wheelhouse, who are not offering themselves for re-election as direct representatives, the Council desires to express its sense of the loss which it will sustain from the absence of these gentlemen, its appreciation of the high services which they have rendered in the past, and its wish that they may enjoy many future years of happiness, usefulness, and prosperity."

